# Methyl 2-(*N*-ethyl­methane­sulfonamido)benzoate

**DOI:** 10.1107/S160053680800007X

**Published:** 2008-01-09

**Authors:** Muhammad Shafiq, M. Nawaz Tahir, Islam Ullah Khan, Waseeq Ahmad Siddiqui, Muhammad Nadeem Arshad

**Affiliations:** a Government College University, Department of Chemistry, Lahore, Pakistan; b University of Sargodha, Department of Physics, Sagrodha, Pakistan

## Abstract

In the mol­ecule of the title compound, C_11_H_15_NO_4_S, the S atom environment is distorted tetrahedral. The methoxy­carbonyl group is oriented at a dihedral angle of 11.8 (2)° with respect to the benzene ring. In the crystal structure, inter­molecular C—H⋯O hydrogen bonds link the mol­ecules into centrosymmetric dimers.

## Related literature

For general background, see: Reissenweber & Mangold (1982 ▶); Mookherjee *et al.* (1989 ▶); Tadashi *et al.* (1982 ▶). For related literature, see: Siddiqui *et al.* (2006 ▶,2007*a* ▶,*b* ▶); Lombardino (1972 ▶); Hanson & Hitchcook (2004 ▶).
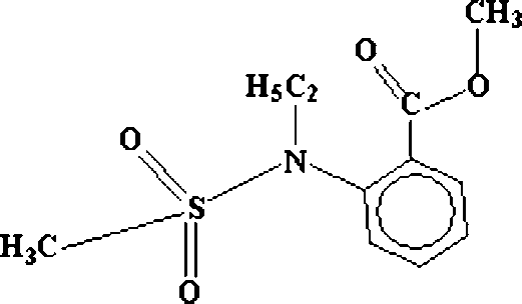


## Experimental

### 

#### Crystal data

C_11_H_15_NO_4_S
*M* *_r_* = 257.30
Triclinic, 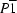

*a* = 8.0161 (4) Å
*b* = 8.4386 (4) Å
*c* = 10.5329 (5) Å
α = 85.244 (3)°
β = 78.721 (3)°
γ = 62.650 (3)°
*V* = 620.61 (5) Å^3^
*Z* = 2
Mo *K*α radiation
μ = 0.26 mm^−1^
*T* = 296 (2) K
0.25 × 0.18 × 0.12 mm

#### Data collection

Bruker Kappa APEXII CCD diffractometer
Absorption correction: multi-scan (*SADABS*; Bruker, 2005) *T*_min_ = 0.935, *T*_max_ = 0.958
11895 measured reflections
3012 independent reflections
1817 reflections with *I* > 3σ(*I*)
*R*_int_ = 0.032

#### Refinement

*R*[*F* ^2^ > 2σ(*F* ^2^)] = 0.043
*wR*(*F* ^2^) = 0.123
*S* = 1.00
3012 reflections
154 parameters
H-atom parameters constrained
Δρ_max_ = 0.37 e Å^−3^
Δρ_min_ = −0.36 e Å^−3^

### 

Data collection: *APEX2* (Bruker, 2007 ▶); cell refinement: *APEX2*; data reduction: *SAINT* (Bruker, 2007 ▶); program(s) used to solve structure: *SHELXS97* (Sheldrick, 2008 ▶); program(s) used to refine structure: *SHELXL97* (Sheldrick, 2008 ▶); molecular graphics: *ORTEP-3 for Windows* (Farrugia, 1997 ▶) and *PLATON* (Spek, 2003 ▶); software used to prepare material for publication: *WinGX* publication routines (Farrugia, 1999 ▶) and *PLATON*.

## Supplementary Material

Crystal structure: contains datablocks global, I. DOI: 10.1107/S160053680800007X/hk2412sup1.cif

Structure factors: contains datablocks I. DOI: 10.1107/S160053680800007X/hk2412Isup2.hkl

Additional supplementary materials:  crystallographic information; 3D view; checkCIF report

## Figures and Tables

**Table:** 

S1—O1	1.421 (2)
S1—O2	1.4303 (19)
S1—N1	1.6269 (18)
S1—C9	1.747 (2)

**Table:** 

O1—S1—O2	119.88 (12)
O1—S1—N1	107.29 (11)
O2—S1—N1	107.34 (11)
O1—S1—C9	108.32 (13)
O2—S1—C9	106.53 (13)
N1—S1—C9	106.83 (11)

**Table 2:** Hydrogen-bond geometry (Å, °)

*D*—H⋯*A*	*D*—H	H⋯*A*	*D*⋯*A*	*D*—H⋯*A*
C9—H9*B*⋯O1^i^	0.96	2.50	3.372 (5)	151
C7—H7*B*⋯O3	0.97	2.57	3.044 (4)	110
C9—H9*C*⋯O3	0.96	2.59	3.152 (4)	117

Symmetry code: (i) 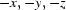.

**Fig. 1.**
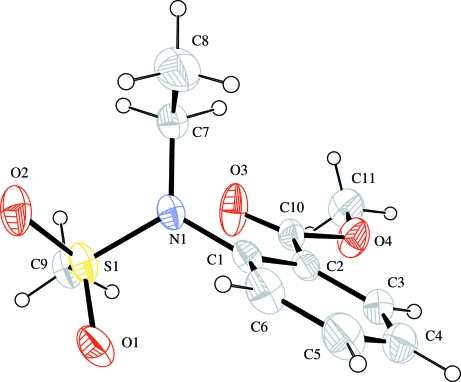
The molecular structure of the title molecule, with the atom-numbering scheme. Displacement ellipsoids are drawn at the 30% probability level.

**Fig. 2.**
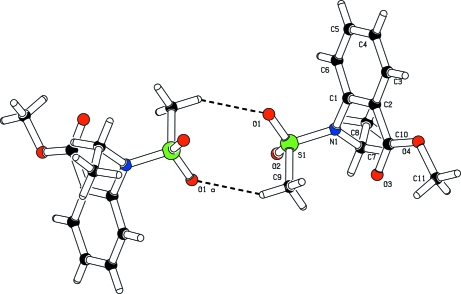
A partial packing diagram of (I). Hydrogen bonds are shown as dashed lines [symmetry code: (a) -x, -y, -z].

## supplementary crystallographic information

### Comment

Alkyl anthranilates are valuable starting materials for the preparation of pesticides, dyes and drugs (Reissenweber & Mangold, 1982). They find organoleptic uses in augmenting the aroma or taste of perfume compositions, colognes, perfumed articles, foodstuffs, medicinal products and in enhancing the effects of deodorancy (Mookherjee *et al.*, 1989). Particularly, the N-substituted thioesters of anthranilic acid have been found potent inhibitors of the serine proteases human leukocyte (HL) elastase, porcine pancreatic elastase, cathepsin G, and bovine chymotrypsin A_α_ (Tadashi *et al.*, 1982). In continuation of our research program to synthesize new biologically important 1,2-benzothiazine 1,1-dioxide molecules (Siddiqui *et al.*, 2006; Siddiqui *et al.*, 2007*a*,b), we embarked on the syntheses of 2,1-benzothiazine 2,2-dioxide, as well. Contrary to the *N*-methyl substituent (Lombardino, 1972), the *N*-ethyl derivative is being reported for the first time.

The title compound, (I), is an important precursor for the synthesis of 2,1-benzothiazine 2,2-dioxide molecule. The structure determination of (I) is undertaken in order to understand the conformational geometry around the sulfur and nitrogen atoms, due to the addition of ethyl group.

In the molecule of (I), (Fig. 1) S1 atom adopts a distorted tetrahedral coordination geometry with two O, one N and one C atoms of methylsulfonyl amino group (Table 1). The sulfur bonds are shortened, while the bond angles aroud it are different with respect to the corresponding values reported in methyl anthranilate *N*-methanesulfonamide, (II), (Hanson & Hitchcook, 2004).

In (I), the addition of ethyl group at N1, instead of H-atom in (II), has changed the orientation of CH_3_ group and O4 atom as compared with (II). On the other hand, the O3—C10 [1.193 (3) Å] and O4—C10 [1.328 (3) Å] bonds in (I), are reported as 1.2202 (16) Å and 1.3324 (16) Å, respectively, in (II). The methyl carboxylate group (O3/O4/C10/C11) is oriented at a dihedral angle of 11.8 (2)° with respect to the phenyl ring (C1—C6).

In the crystal structure, intermolecular C—H···O hydrogen bonds (Table 2) link the molecules into centrosymmetric dimers (Fig. 2), in which they may be effective in the stabilization of the structure.

### Experimental

For the preparation of the title compound, the suspension of hexane-washed sodium hydride (50% in mineral oil) was prepared in dry dimethylformamide (3 ml). A solution of methyl *N*-methylsulfonylanthranilate (70 mg, 0.306 mmol) in dry dimethylformamide (5 ml) was added to the suspension (76 mg, 0.368 mmol), and stirred for 45 min at room temperature. Then, a solution of ethyl iodide (144 mg, 0.92 mmol) in ether (5 ml) was added to it. The resulting white suspension was stirred for 1.5 h and poured into hydrochloric acid (3 N, 50 ml) to produce a yellow suspension which was extracted with chloroform (4 × 25 ml). The combined extract was dried over calcium sulfate and evaporated under reduced pressure (11 torr) to get the title compound (yield; 58 mg, 73%, m.p. 330–331 K). Crystals suitable for X-ray analysis were obtained by slow evaporation of CHCl_3_.

### Refinement

H atoms were positioned geometrically, with C—H = 0.93, 0.97 and 0.96 Å for aromatic, methylene and methyl H, and constrained to ride on their parent atoms, with *U*_iso_(H) = *xU*_eq_(C), where *x* = 1.5 for methyl H, and *x* = 1.2 for all other H atoms.

### Crystal data

**Table:**

C_11_H_15_NO_4_S	*Z* = 2
*M**_r_* = 257.30	*F*_000_ = 272
Triclinic, *P*1	*D*_x_ = 1.377 Mg m^−^^3^
Hall symbol: -P 1	Mo *K*α radiation λ = 0.71073 Å
*a* = 8.0161 (4) Å	Cell parameters from 3596 reflections
*b* = 8.4386 (4) Å	θ = 2.7–24.8º
*c* = 10.5329 (5) Å	µ = 0.26 mm^−^^1^
α = 85.244 (3)º	*T* = 296 (2) K
β = 78.721 (3)º	Prismatic, colourless
γ = 62.650 (3)º	0.25 × 0.18 × 0.12 mm
*V* = 620.61 (5) Å^3^	

### Data collection

**Table:**

Bruker KAPPA APEXII CCD diffractometer	3012 independent reflections
Radiation source: fine-focus sealed tube	1817 reflections with *I* > 3σ(*I*)
Monochromator: graphite	*R*_int_ = 0.032
Detector resolution: 8.33 pixels mm^-1^	θ_max_ = 28.3º
*T* = 296(2) K	θ_min_ = 2.0º
ω scans	*h* = −10→10
Absorption correction: multi-scan(SADABS; Bruker, 2005)	*k* = −11→11
*T*_min_ = 0.935, *T*_max_ = 0.958	*l* = −14→14
11895 measured reflections	

### Refinement

**Table:**

Refinement on *F*^2^	Secondary atom site location: difference Fourier map
Least-squares matrix: full	Hydrogen site location: inferred from neighbouring sites
*R*[*F*^2^ > 2σ(*F*^2^)] = 0.043	H-atom parameters constrained
*wR*(*F*^2^) = 0.123	*w* = 1/[σ^2^(*F*_o_^2^) + (0.0727*P*)^2^ + 0.1532*P*] where *P* = (*F*_o_^2^ + 2*F*_c_^2^)/3
*S* = 1.00	(Δ/σ)_max_ < 0.001
3012 reflections	Δρ_max_ = 0.37 e Å^−^^3^
154 parameters	Δρ_min_ = −0.36 e Å^−^^3^
Primary atom site location: structure-invariant direct methods	Extinction correction: none

### Special details

**Table:**

Geometry. All e.s.d.'s (except the e.s.d. in the dihedral angle between two l.s. planes) are estimated using the full covariance matrix. The cell e.s.d.'s are taken into account individually in the estimation of e.s.d.'s in distances, angles and torsion angles; correlations between e.s.d.'s in cell parameters are only used when they are defined by crystal symmetry. An approximate (isotropic) treatment of cell e.s.d.'s is used for estimating e.s.d.'s involving l.s. planes.
Refinement. Refinement of *F*^2^ against ALL reflections. The weighted *R*-factor *wR* and goodness of fit *S* are based on *F*^2^, conventional *R*-factors *R* are based on *F*, with *F* set to zero for negative *F*^2^. The threshold expression of *F*^2^ > σ(*F*^2^) is used only for calculating *R*-factors(gt) *etc*. and is not relevant to the choice of reflections for refinement. *R*-factors based on *F*^2^ are statistically about twice as large as those based on *F*, and *R*- factors based on ALL data will be even larger.

### Fractional atomic coordinates and isotropic or equivalent isotropic displacement parameters (Å^2^)

**Table:**

	*x*	*y*	*z*	*U*_iso_*/*U*_eq_	
S1	0.14595 (10)	0.13878 (8)	0.07989 (5)	0.0507 (2)	
O1	−0.0510 (3)	0.1954 (3)	0.08167 (19)	0.0688 (6)	
O2	0.2655 (3)	0.1495 (3)	−0.03718 (16)	0.0707 (6)	
O3	0.2630 (3)	0.0251 (3)	0.40332 (17)	0.0811 (7)	
O4	0.0983 (3)	0.1413 (3)	0.59441 (16)	0.0700 (6)	
N1	0.1604 (3)	0.2545 (3)	0.18962 (17)	0.0457 (5)	
C1	−0.0041 (3)	0.3538 (3)	0.2846 (2)	0.0443 (6)	
C2	−0.0200 (3)	0.3048 (3)	0.4158 (2)	0.0436 (6)	
C3	−0.1825 (4)	0.4142 (4)	0.5006 (3)	0.0605 (7)	
H3	−0.195	0.3845	0.5878	0.073*	
C4	−0.3251 (4)	0.5646 (4)	0.4595 (4)	0.0758 (9)	
H4	−0.4337	0.6344	0.5181	0.091*	
C5	−0.3076 (5)	0.6121 (4)	0.3320 (4)	0.0801 (10)	
H5	−0.4039	0.7146	0.3039	0.096*	
C6	−0.1476 (4)	0.5080 (4)	0.2458 (3)	0.0654 (8)	
H6	−0.1356	0.542	0.1596	0.078*	
C7	0.3401 (4)	0.2613 (4)	0.1897 (2)	0.0565 (7)	
H7A	0.4445	0.1594	0.1417	0.068*	
H7B	0.3614	0.2524	0.2781	0.068*	
C8	0.3412 (5)	0.4294 (5)	0.1310 (3)	0.0872 (11)	
H8A	0.4615	0.427	0.1332	0.131*	
H8B	0.2402	0.5307	0.1794	0.131*	
H8C	0.3228	0.4378	0.0429	0.131*	
C9	0.2485 (4)	−0.0838 (3)	0.1283 (2)	0.0573 (7)	
H9A	0.3819	−0.1245	0.1276	0.086*	
H9B	0.2333	−0.1556	0.0697	0.086*	
H9C	0.1866	−0.0938	0.2141	0.086*	
C10	0.1286 (4)	0.1421 (3)	0.4659 (2)	0.0457 (6)	
C11	0.2403 (5)	−0.0050 (4)	0.6546 (3)	0.0754 (9)	
H11A	0.204	0.0075	0.747	0.113*	
H11B	0.3615	−0.0042	0.6282	0.113*	
H11C	0.2499	−0.1157	0.6285	0.113*	

### Atomic displacement parameters (Å^2^)

**Table:**

	*U*^11^	*U*^22^	*U*^33^	*U*^12^	*U*^13^	*U*^23^
S1	0.0718 (5)	0.0469 (4)	0.0342 (3)	−0.0235 (3)	−0.0201 (3)	0.0011 (2)
O1	0.0720 (13)	0.0634 (12)	0.0718 (12)	−0.0208 (10)	−0.0347 (10)	−0.0126 (9)
O2	0.1094 (17)	0.0694 (13)	0.0324 (9)	−0.0411 (12)	−0.0106 (9)	0.0037 (8)
O3	0.0977 (16)	0.0587 (12)	0.0408 (10)	0.0047 (11)	−0.0141 (10)	−0.0030 (9)
O4	0.0661 (13)	0.0920 (15)	0.0355 (9)	−0.0220 (11)	−0.0101 (9)	0.0021 (9)
N1	0.0579 (13)	0.0471 (11)	0.0337 (9)	−0.0239 (10)	−0.0109 (9)	−0.0018 (8)
C1	0.0527 (15)	0.0365 (12)	0.0475 (12)	−0.0200 (11)	−0.0169 (11)	−0.0023 (9)
C2	0.0484 (14)	0.0446 (13)	0.0417 (12)	−0.0224 (12)	−0.0104 (11)	−0.0062 (10)
C3	0.0547 (17)	0.0681 (18)	0.0569 (15)	−0.0256 (15)	−0.0035 (13)	−0.0186 (13)
C4	0.0540 (19)	0.065 (2)	0.095 (2)	−0.0127 (16)	−0.0083 (17)	−0.0345 (17)
C5	0.067 (2)	0.0508 (17)	0.106 (3)	−0.0015 (15)	−0.038 (2)	−0.0169 (17)
C6	0.079 (2)	0.0450 (15)	0.0677 (17)	−0.0174 (15)	−0.0307 (16)	0.0023 (12)
C7	0.0632 (17)	0.0686 (17)	0.0439 (13)	−0.0347 (15)	−0.0087 (12)	−0.0046 (12)
C8	0.122 (3)	0.094 (3)	0.075 (2)	−0.077 (2)	−0.013 (2)	0.0089 (18)
C9	0.0779 (19)	0.0455 (14)	0.0483 (14)	−0.0252 (14)	−0.0173 (13)	−0.0010 (11)
C10	0.0556 (16)	0.0491 (14)	0.0348 (11)	−0.0255 (13)	−0.0077 (11)	−0.0016 (10)
C11	0.082 (2)	0.095 (2)	0.0440 (14)	−0.0339 (18)	−0.0227 (15)	0.0166 (14)

### Geometric parameters (Å, °)

**Table:**

S1—O1	1.421 (2)	C4—H4	0.93
S1—O2	1.4303 (19)	C5—C6	1.372 (4)
S1—N1	1.6269 (18)	C5—H5	0.93
S1—C9	1.747 (2)	C6—H6	0.93
O3—C10	1.193 (3)	C7—C8	1.503 (4)
O4—C10	1.328 (3)	C7—H7A	0.97
O4—C11	1.443 (3)	C7—H7B	0.97
N1—C1	1.431 (3)	C8—H8A	0.96
N1—C7	1.468 (3)	C8—H8B	0.96
C1—C6	1.380 (3)	C8—H8C	0.96
C1—C2	1.408 (3)	C9—H9A	0.96
C2—C3	1.387 (3)	C9—H9B	0.96
C2—C10	1.485 (3)	C9—H9C	0.96
C3—C4	1.369 (4)	C11—H11A	0.96
C3—H3	0.93	C11—H11B	0.96
C4—C5	1.368 (5)	C11—H11C	0.96
			
O1—S1—O2	119.88 (12)	N1—C7—C8	112.8 (2)
O1—S1—N1	107.29 (11)	N1—C7—H7A	109
O2—S1—N1	107.34 (11)	C8—C7—H7A	109
O1—S1—C9	108.32 (13)	N1—C7—H7B	109
O2—S1—C9	106.53 (13)	C8—C7—H7B	109
N1—S1—C9	106.83 (11)	H7A—C7—H7B	107.8
C10—O4—C11	116.5 (2)	C7—C8—H8A	109.5
C1—N1—C7	119.82 (18)	C7—C8—H8B	109.5
C1—N1—S1	119.87 (16)	H8A—C8—H8B	109.5
C7—N1—S1	120.29 (16)	C7—C8—H8C	109.5
C6—C1—C2	119.3 (2)	H8A—C8—H8C	109.5
C6—C1—N1	118.0 (2)	H8B—C8—H8C	109.5
C2—C1—N1	122.6 (2)	S1—C9—H9A	109.5
C3—C2—C1	117.9 (2)	S1—C9—H9B	109.5
C3—C2—C10	119.3 (2)	H9A—C9—H9B	109.5
C1—C2—C10	122.8 (2)	S1—C9—H9C	109.5
C4—C3—C2	121.8 (3)	H9A—C9—H9C	109.5
C4—C3—H3	119.1	H9B—C9—H9C	109.5
C2—C3—H3	119.1	O3—C10—O4	121.8 (2)
C5—C4—C3	119.9 (3)	O3—C10—C2	126.8 (2)
C5—C4—H4	120	O4—C10—C2	111.4 (2)
C3—C4—H4	120	O4—C11—H11A	109.5
C4—C5—C6	119.7 (3)	O4—C11—H11B	109.5
C4—C5—H5	120.1	H11A—C11—H11B	109.5
C6—C5—H5	120.1	O4—C11—H11C	109.5
C5—C6—C1	121.3 (3)	H11A—C11—H11C	109.5
C5—C6—H6	119.3	H11B—C11—H11C	109.5
C1—C6—H6	119.3		
			
O1—S1—N1—C1	14.2 (2)	C10—C2—C3—C4	−179.2 (2)
O2—S1—N1—C1	144.26 (18)	C2—C3—C4—C5	−1.2 (4)
C9—S1—N1—C1	−101.8 (2)	C3—C4—C5—C6	0.4 (5)
O1—S1—N1—C7	−164.23 (18)	C4—C5—C6—C1	1.0 (5)
O2—S1—N1—C7	−34.2 (2)	C2—C1—C6—C5	−1.7 (4)
C9—S1—N1—C7	79.8 (2)	N1—C1—C6—C5	−178.6 (3)
C7—N1—C1—C6	104.8 (3)	C1—N1—C7—C8	−78.2 (3)
S1—N1—C1—C6	−73.6 (3)	S1—N1—C7—C8	100.3 (2)
C7—N1—C1—C2	−72.0 (3)	C11—O4—C10—O3	2.3 (4)
S1—N1—C1—C2	109.6 (2)	C11—O4—C10—C2	−176.2 (2)
C6—C1—C2—C3	0.9 (3)	C3—C2—C10—O3	170.1 (3)
N1—C1—C2—C3	177.6 (2)	C1—C2—C10—O3	−9.6 (4)
C6—C1—C2—C10	−179.4 (2)	C3—C2—C10—O4	−11.5 (3)
N1—C1—C2—C10	−2.7 (3)	C1—C2—C10—O4	168.9 (2)
C1—C2—C3—C4	0.5 (4)		

### Hydrogen-bond geometry (Å, °)

**Table:**

*D*—H···*A*	*D*—H	H···*A*	*D*···*A*	*D*—H···*A*
C9—H9B···O1^i^	0.96	2.50	3.372 (5)	151
C7—H7B···O3	0.97	2.57	3.044 (4)	110
C9—H9C···O3	0.96	2.59	3.152 (4)	117

Symmetry codes: (i) −*x*, −*y*, −*z*.
